# Gene Therapy for Pancreatic Diseases: Current Status

**DOI:** 10.3390/ijms19113415

**Published:** 2018-10-31

**Authors:** Kenya Kamimura, Takeshi Yokoo, Shuji Terai

**Affiliations:** Division of Gastroenterology and Hepatology, Graduate School of Medical and Dental Sciences, Niigata University, 1-757, Aasahimachi-Dori, Chuo-Ku, Niigata 951-8510, Japan; t-yokoo@med.niigata-u.ac.jp (T.Y.); terais@med.niigata-u.ac.jp (S.T.)

**Keywords:** gene therapy, pancreas, cancer, pancreatitis, diabetes mellitus, pain

## Abstract

The pancreas is a key organ involved in digestion and endocrine functions in the body. The major diseases of the pancreas include pancreatitis, pancreatic cancer, cystic diseases, pancreatic divisum, islet cell tumors, endocrine tumors, diabetes mellitus, and pancreatic pain induced by these diseases. While various therapeutic methodologies have been established to date, however, the improvement of conventional treatments and establishment of novel therapies are essential to improve the efficacy. For example, conventional therapeutic options, including chemotherapy, are not effective against pancreatic cancer, and despite improvements in the last decade, the mortality rate has not declined and is estimated to become the second cause of cancer-related deaths by 2030. Therefore, continuous efforts focus on the development of novel therapeutic options. In this review, we will summarize the progress toward the development of gene therapies for pancreatic diseases, with an emphasis on recent preclinical studies and clinical trials. We aim to identify new areas for improvement of the current methodologies and new strategies that will lead to safe and effective gene therapeutic approaches in pancreatic diseases.

## 1. Introduction

The pancreas is a key organ involved in digestion and endocrine functions, located in the retroperitoneal space, and measures approximately 15–20 cm in length. The pancreatic duct, that runs along the organ, connects to the duodenum. One major function of the pancreas is digestion of proteins, fats, and carbohydrates by enzymes released through the duct. A second important role of the pancreas is endocrine activity mediated by several hormones, such as insulin, released from the pancreatic glands to the blood circulation, which help maintain the homeostasis in the body. Major diseases of the pancreas include pancreatitis, pancreatic cancer, cystic diseases, pancreatic divisum, islet cell tumors, endocrine tumors, and diabetes mellitus [1].

Among the pathologies involving the pancreas, pancreatic cancer is one of the leading causes of the cancer-related deaths globally and currently is the fourth cause of cancer-related mortalities in the United States [2]. Pancreatic ductal adenocarcinoma, the most common histological type, has a poor prognosis and aggressive growth pattern [3]. Although poor glycemic control and weight loss in patients with long-standing diabetes were proposed as useful markers for pancreatic cancer [4], there are currently no markers or strategies that can detect the tumor in the early phase. In addition, although depletion of adipose and skeletal muscle tissues in the early stages of cancer is common, recent evidence suggests that early loss of peripheral tissue in association with pancreatic cancer may not impair survival [5]; therefore, a better understanding of the mechanism underlying the progression of this aggressive cancer is essential. Recent epidemiological studies clearly show that the mortality rate of pancreatic cancer is not declining, and pancreatic cancer is estimated to become the second cause of cancer-related deaths by 2030 [6]. Therefore, significant effort is spent on the development of novel therapeutic options for this devastating disease.

Although several novel chemotherapeutic compounds, including nab-paclitaxel and liposomal irinotecan, and chemotherapy regimens, such as FOLFIRINOX, have been successfully tested in clinical trials as conventional treatment options [7], the prognosis remains poor [8]. To improve the prognosis of pancreatic cancer, the number of basic researches and clinical trials are increasing [7]. In addition, the comprehensive precision medicine tools for therapeutic options that have been implemented in both the community and academic areas [9] have led to better progression-free survival rates in patients receiving matched therapy.

In a disease where novel therapeutic innovations are sorely needed, combining novel therapies, including gene and cell therapy approaches, with the currently available cytotoxic chemotherapeutic drugs and radiation therapy, provides hope for better outcomes in patients with advanced-stage pancreatic cancer. Among such potentially innovative treatment approaches, gene therapy offers a promising avenue based on the results of several completed and ongoing clinical trials. The summaries of these studies are shown in Table 1 and Table 2.

Preclinical studies have led to a better understanding of pancreatic cancer, as well as potential therapeutic target genes, including tumor suppressor genes, antiangiogenic and pro-apoptotic genes, and suicide genes [10]. A study including 3030 patients with pancreatic cancer showed significant associations with germline mutations in *CDKN2A*, *TP53*, *MLH1*, *BRCA2*, *ATM*, and *BRCA1* and the risk of the cancer occurrence [11]. Similar results have been reported in studies focusing on the role of *KRAS* gene in tumor development [12], and research on inactivation of the DNA maintenance genes, including *BRCA1*, *BRCA2*, and *PALB2*, demonstrated that patients with pancreatic cancer with defective DNA maintenance genes responded better to platinum-based chemotherapy [13]. In addition, preclinical studies led to the identification of potential therapeutic target genes. For example, downregulation of the microRNA, miR-98-5p promoted pancreatic tumor development by downregulation of MAP4K4 and inhibition of the downstream MAPK/ERK signaling, suggesting that the miR-98-5p can be a therapeutic target in pancreatic cancer treatment [14]. Similarly, spalt-like protein 4 induced endothelial-mesenchymal transitions to facilitate metastatic potential in pancreatic ductal adenocarcinoma (PDAC) cells, indicating that spalt-like protein 4 might be a marker for PDAC treatment and that targeting this protein might benefit anti-proliferative and anti-metastasis therapies [15]. A study demonstrated that the knockout of epithelial cell transforming 2 gene by the clustered regularly interspaced short palindromic repeats/CRISPR associated protein 9 (CRISPR/Cas9) gene editing method decreased proliferation and ability to migrate in the MiaPaCa2 pancreatic cancer cell line [16]. Furthermore, three intratumoral infiltrating immune markers (CD15, CD206 and CD117) and one *SMAD4* mutation were found to be associated with recurrence and survival in patients after surgery for PDAC [17] and G protein alpha-driven oncogenic mechanisms, Siks revealed unanticipated metabolic heterogeneity among *KRAS*-mutant pancreatic neoplasms and evidenced to be a potent tumor suppressor [18].

Other preclinical studies, however, demonstrated the relationship of chemotherapy and gene expression [48]. A study showing gemcitabine-mediated tumor regression and p53-dependent gene expression increased the possibility that targeting the Bax-dependent cell death pathway, rather than the PUMA pathway, might result in significantly improved patient outcomes and prognosis in pancreatic cancer [49]. Intravenous administration of Rexin-G^TM^, a nonreplicative pathology-targeted retroviral vector bearing a cytocidal cyclin G1 construct, exhibited a good safety and efficacy profile in a phase I/II trial of gemcitabine-resistant pancreatic cancer [30]. Intratumoral administration of CYL-02, a nonviral gene therapy product, using endoscopic ultrasound was found to be safe in a phase I trial including 22 patients who were concomitantly treated with gemcitabine [44]. Conversely, RNAi therapy targeting *KRAS* in combination with chemotherapy showed efficacy in treating locally advanced pancreatic cancer patients [40], whereas the G-protein-coupled receptor family C, member 5, group A gene was upregulated in PaCa cells, which led to enhanced drug resistance in PaCa cells [50]. These data can aid in the implementation of personalized therapy for improved outcomes in patients with pancreatic cancer. In addition to pancreatic malignancies, ongoing studies are aimed to establish effective gene therapies for pancreatitis, pancreatic pain, and diabetes mellitus. Based on the several completed (Table 1) and ongoing clinical trials [19,20,21,22,23,24,25,26,27,28,29,30,31,32,33,34,35,36,37,38,39,40,41,42,43,44,45,46,47] (Table 2), gene therapy offers a promising avenue as a safe and efficient approach [51].

Various techniques used for gene therapy, including synthetic and viral vectors, molecular tools, such as interference RNA, and genome editing methods, showed promising results in filling the gap between experimental models of cancer and clinical trials [10,52], therefore in this review, the methods of gene delivery into the pancreas used in studies are also summarized (Table 3). This review summarizes the current landscape of completed and ongoing studies on these diseases for both the physicians and the researchers.

## 2. Pancreatic Cancer

The latest developments in pancreatic carcinoma gene therapy include gene-based tumor cell sensitization to chemotherapy, vaccination, and adoptive immunotherapy (chimeric antigen receptor T-cell [CAR-T] therapy). Furthermore, oncolytic virus therapies, including oncolytic adenoviruses, herpes virus, parvovirus, and reovirus are currently in development [10].

Preclinical studies showed promising results in the efficacy of gene therapy. For example, the Lister strain of vaccinia virus armed with the endostatin-angiostatin fusion gene exhibited therapeutic efficacy for potential pancreatic cancer [53], whereas the inhibition of miR-21 led to the inhibition of pancreatic cancer tumor growth both in vitro and in vivo [54]. Additionally, introduction of decoy hyper-binding sites to sequester and suppress the entry of oncogenic HMGA1 into the nucleus of pancreatic cancer cells by replication-defective adenovirus serotype 5 virus achieved inhibition of cancer cell growth [55].

Virus vectors used in these gene therapy studies include adeno-associated virus (AAV) vectors with capsids that are modified and optimized by site-directed mutagenesis to improve the transduction efficiency in the pancreatic tissue, especially in pancreatic cancer cells [56]. Studies using oncolytic virus vectors utilize hexon modification to improve the activity of the oncolytic adenovirus vectors targeting the TGFBR to enable replication in tumor stromal cells as well [57]. Targeted herpes simplex virus (HSV)-1 (HSV-1) whose expression is regulated by the cellular B-myb promoter in an experimental model of pancreatic tumor resulted in an effective reduction in tumor growth [58]. However, in a phase II clinical trial, the progression-free survival rate was not improved in patients with metastatic pancreatic cancer who were treated with chemotherapy in combination with the oncolytic virus pelareorep, which causes oncolysis in pancreatic cells by activating Ras [42]. On the other hand, oncosuppressive parvovirus H1 increased sensitivity to chemotherapy and inhibited the activation of the NFκB and the Akt/PI3K pathways in patients with pancreatic peritoneal carcinomatosis [59].

Recent advances in the safety and efficacy of the CRISPR/Cas9 genome editing method [60] led to several studies that investigated its utility in pancreatic cancer. For example, retrograde pancreatic ductal injection of adenoviral-Cre and lentiviral-Cre vectors were utilized to develop in vivo models of pancreatic cancer [61]; this technique can significantly contribute to the establishment of novel therapies for pancreatic cancer.

Additional strategies utilizing gene targeting methods include cell therapies, such as T-cell programming [62], immunotherapy, and CAR-T [63,64]; induction of T-cell immunity to cancer antigens, such as mesothelin by GVAX pancreas; and granulocyte-macrophage colony-stimulating factor-secreting allogeneic pancreatic tumor cells for pancreatic cancer patients with metastatic lesions [41].

These studies are likely to change the clinical practice and approaches to PDAC management, as they represent a major advancement not only for PDAC, but also for the broader field of gene-based molecular treatment of cancer.

## 3. Pancreatitis

Pancreatitis, inflammation of the pancreas, can be classified into acute and chronic forms. Inflammation can be due to various factors, including alcohol, fatty food, gallstones, high serum calcium concentrations caused by the hyperparathyroidism, abdominal injury, chemotherapeutic agents, and pancreatic neoplasms. The etiological factor activates the pancreatic digestive enzymes in the pancreas itself instead of the small intestine. Abdominal pain, back pain, fever, nausea, and diarrhea are the major symptoms of pancreatitis, which may lead to abscess formation, infection, pseudocysts, multiple organ failure, malnutrition, diabetes mellitus, and pancreatic cancer.

Among the various etiologies, associations of genetic mutations with acute recurrent and chronic pancreatitis in children were reported [65]. And gene therapy was studied in patients with genetic lipoprotein lipase deficiency-induced pancreatitis [66]. Alipogene tiparvovec was tested in 19 patients with lipoprotein lipase deficiency, and the retrospective analysis of follow-up for up to 6 years after a single treatment demonstrated the association of alipogene tiparvovec with lower frequency and severity of pancreatitis events [66]. In addition, the genetic association of recurrent autoimmune pancreatitis with resistance to steroid therapy is currently evaluated with NCT00444444. In this study, high-resolution typing for HLA with sequence-based typing is utilized to determine whether certain alleles or haplotypes of major histocompatibility complex gene are associated with autoimmune pancreatitis. Further analyses and trials will be conducted with the genomic information of this disease entity.

## 4. Pancreatic Pain

Abdominal pain is a major symptom associated with pancreatic diseases, and improvement of pain in acute pancreatitis is associated with the resolution of inflammation; however, pain related to pancreatic cancer and chronic pancreatitis persists and recurs and is therefore difficult to manage [67]. The currently available management methods include analgesics, antioxidant micronutrients, drugs interfering with neural transmission, pancreatic enzymes, celiac plexus blockade, and surgery [67]. As the severity of pain is associated with a decrease in quality of life, loss of appetite, and longer and frequent hospitalization and given that the development of various chemotherapeutic options for pancreatic cancer have led to a longer lifespan, it is essential to develop novel therapeutic options to manage pancreatic pain. Recent preclinical studies revealed that several neuroimmune and neuroplastic mechanisms, including infiltration of inflammatory cells, neural edema, perineural disruption, and neural hypertrophy, are involved as the underlying mechanisms of chronic pancreatitis pain [67]. Furthermore, a recent study reported the contribution of the neuronal transforming growth factor beta (TGF-β) signaling mediated via neuronal SMAD3 to the development of pancreatic pain in an animal model [68].

Based on these findings, a replication-defective herpes viral vector (HSV-1) construct encoding the human preproenkephalin (*ENK*) gene (HSV-ENK) was tested in a preclinical in vivo study [69,70]. HSV-ENK was applied directly to the surface of the pancreas in rats with dibutyltin dichloride-induced pancreatitis rats, and their spontaneous behavioral activity was monitored. Compared with the controls, HSV-ENK-treated rats showed significantly increased expression of *ENK* in the pancreas, improved activity, and normalized FOS staining, indicating reduced neuronal activity [69]. Similar results were shown in rat models of high-fat- and alcohol liquid diet-induced pancreatitis [70]. Importantly, both studies showed the preservation of the acinar cells and amelioration of the inflammation, suggesting that this method might be potentially effective for not only pain control, but also pancreatic function in chronic pancreatitis.

The overexpression of TGF-β in mice and rats was associated with sensory neuron hyperexcitability, SMAD3 activation, and increase in nociception. As chronic pancreatitis leads to pancreatic fibrosis and that the TGF-β signaling pathway was shown to be associated with fibrosis, this signaling pathway can be utilized as a therapeutic target to reduce pain and fibrosis in patients with chronic pancreatitis [68]. Given that the currently available treatment options are not sufficient to manage the severe pancreatic pain, development of new strategies are sorely needed.

## 5. Diabetes Mellitus

Diabetes mellitus is characterized by hyperglycemia arising from dysregulation of insulin and categorized into type 1, type 2, and gestational diabetes. The treatment approaches in diabetes depend on the type. Insulin replacement is used to treat patients with type 1 diabetes, which is caused by the impairment of pancreatic beta cells producing insulin. Type 2 diabetes is caused by an insufficient insulin level or hyposensitivity of the cells in the body to insulin, whereas gestational diabetes occurs in females during pregnancy and requires careful management to avoid complications in the child. Since chronic hyperglycemia leads dysfunction in various organs, including the vessels, heart, kidneys, retina, and the nervous system, careful management of blood glucose levels is necessary.

Genetic predisposition to diabetes has been reported in several studies. For example, mutation of neuronal differentiation 1, a basic helix-loop-helix transcription factor involved in the development of pancreas, can be found in patients with neonatal diabetes, as well as those with type 2 diabetes mellitus [71]. Gene therapy approaches currently explored for diabetes mellitus include insulin gene therapy for type 1 diabetes mellitus, in which insulin expression in non-beta cells of the pancreas with hepatocytes emerged as the primary therapeutic target [72]; fibroblast growth factor 21 (FGF21) gene therapy, which has showed promising results in a mouse model of metabolic disease [73]; GLP-1 gene therapy, which improved insulin sensitivity and reduced abdominal and hepatic fat associated with obesity-induced diabetes mellitus and altered adipokine profiles [74]; and in vivo reprogramming of pancreatic ductal cells by intraductal delivery of an adenoviral vector expressing transcription factors essential for beta cell function, which resulted in the induction of beta-like cells and correction of hyperglycemia [75]. The combination of gene and cell therapy has also been reported to induce insulin-producing cells from the hepatocytes of newborn rats. In that study, analysis of miRNA expression the differentiation of hepatocytes to insulin-producing cells was associated with the BMP/Smad signaling pathway rather than the Wnt/β-catenin signaling pathway [76]. More recently, transfection of Sarco-ER Ca^2+^-ATPase 2b gene in adipose mesenchymal stem cells exhibited a significant therapeutic effect in a mouse model of type 1 diabetes [77]. Recent reports also showed the effect of FGF21 gene therapy on a high-fat-diet mouse model where treatment was associated with significant reductions in body weight, adipose tissue hypertrophy, insulin resistance, hepatic steatosis, inflammation, and fibrotic changes [78]. That study used AAV vectors to mediate long-term and sustained protein production using liver-specific promoters, which resulted in sustained levels of circulating FGF21 for up to 1 year. Interestingly, FGF21 overexpression in normal mice fed with standard diet was also associated with the prevention of weight gain and insulin resistance related to aging [78]. These preclinical studies illustrate the promising results of gene therapy for diabetes by targeting the pancreas.

## 6. Delivery Methods for Pancreatic Gene Therapy

**Figure 1:**
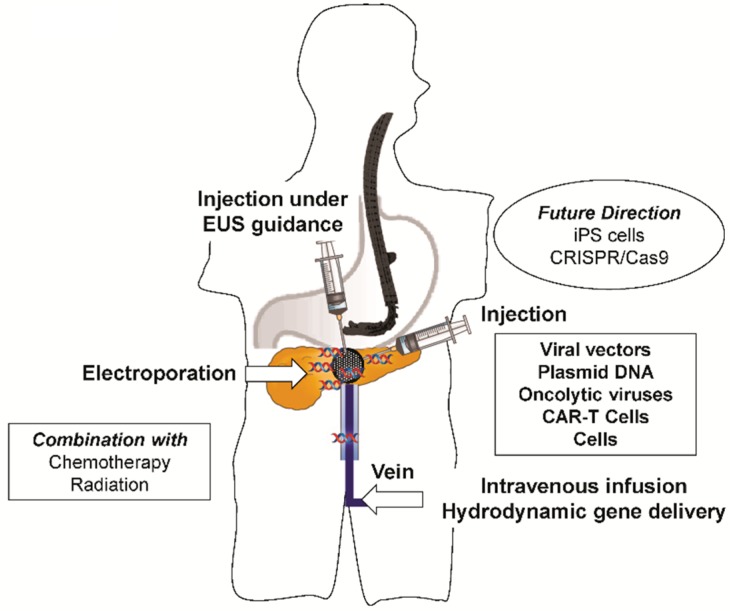
Schematic summary of the strategies of gene therapy for the pancreas. EUS, endoscopic ultrasound; iPS cells, inducible pluripotent stem cells; CRISPR/Cas9, clustered regularly interspaced short palindromic repeats/CRISPR associated protein 9; CAR-T, chimeric antigen receptor-T cell; DNA, deoxyribonucleic acid.

The various methods for gene delivery used in clinical and preclinical studies of the pancreas can be classified into ex vivo and in vivo methods [79]. Ex vivo gene transfer involves genetic modification of the cells harvested from the patients or experimental animals, which are then reintroduced to the patients or the animals. In vivo gene delivery methods can be further categorized into systemic administration and in situ delivery. The strategies of gene therapy for the pancreas are summarized in Figure 1.

Viral vectors used in studies thus far include oncoretrovirus, lentivirus, foamy virus, adenovirus, AAV, and HSV (Table 1 and Table 2). The features of these viral vectors are summarized in Table 3. Among these, the adenovirus and AAV vectors are commonly used as gene carriers in clinical trials (Table 1 and Table 2). For example, an ongoing phase III trial (NCT00051467) is assessing whether TNFerade, a replication-deficient adenovirus vector that delivers tumor necrosis factor-alpha to tumor cells and is controlled by a chemoradiation-inducible promoter, in combination with 5-fluorouracil and radiation therapy will enhance their anti-tumor effect as first-line treatment of unresectable locally advanced pancreatic cancer.

For in vivo gene delivery, non-viral vector-based methods, including plasmid DNA injection, have also been utilized to deliver genes into target sites in the pancreas in clinical trials (Table 1 and Table 2), whereas other non-viral vector-based methods, including chemical compound carriers and physical methods of gene delivery, have been tested in preclinical trials, as described earlier. Among the chemical approaches are cationic lipids, polymers, and chemically modified proteins, whereas simple needle injection, gene gun, electroporation, sonoporation, magnetofection, and hydrodynamic gene delivery are major methods of delivery by physical methods. The features of the gene delivery methods are also summarized in Table 3. In addition to the currently ongoing clinical trials assessing plasmid gene injection, as well as viral vectors, a study (NCT02514421) is evaluating the efficacy and safety of electrochemotherapy using electroporation for the treatment of pancreatic cancer. Overall, these studies demonstrated the significance of the physical approach in clinical application. Furthermore, image-based guidance is essential for site-specific gene delivery in vivo, especially in the pancreas. Therefore, combination of gene delivery approaches with endoscopic ultrasound has been tested their safe and effective in situ delivery in pancreatic cancer [20,23,25,27,31,33,34,37] (Table 1 and Table 2). This technique has been successfully used in a clinic setting to block sympathetic innervation for the management of chronic pancreatic pain, wherein endoscopic ultrasound is used to inject neurolytic solutions to celiac plexus for neurolysis; this technique can be used to deliver genes. In the currently ongoing clinical trials, this technique has been combined with intratumoral injection of viral vectors, plasmids, and immunologically modified cells, among others (Table 3).

A recent preclinical study by Kamimura et al. reported pancreas-targeted and specific hydrodynamic gene delivery through the superior mesenteric vein into the pancreas and demonstrated its efficacy and safety using naked DNA [80]. Although the insertion of the catheter is essential, this clinically well-stablished physical and organ-specific gene delivery method can be used for both gene therapy studies and the establishment of pancreatic cancer animal models, in which oncogenic genes can be delivered to the pancreas to study various pancreatic tumor types for the development of appropriate therapeutic strategies. Another approach of siRNA delivery is ionizable low-molecular-weight polymeric nanoparticles, which has been demonstrated in nonhuman primates [81]. That study showing endothelial gene silencing in multiple nonhuman primate organs using systemically administered siRNA nanoparticles highlights the potential of this approach for the treatment of disease in humans. Recent report showed that the autophagy is related to pancreatic cancer development and the disruption of the autophagy related gene and reduced autophagy related protein level contributed to inhibit the tumorigenesis. Therefore, the gene therapy managing the autophagy might be the additional option and the nucleic acids to control the gene expression is promising strategy [82]. Further efforts are essential to develop and improve appropriate gene delivery methods for pancreatic diseases.

## 7. Clinical Trials

Building on preclinical studies, several clinical trials have been conducted to evaluate gene therapy in pancreatic diseases, especially pancreatic cancer [19,20,21,22,23,24,25,26,27,28,29,30,31,32,33,34,35,36,37,38,39,40,41,42,43,44,45,46,47]. A summary of these studies can be found in Table 1. Adenoviruses, plasmids, and synthetic vectors were used to deliver interleukin and suicide genes for therapeutic purposes. These genes were delivered by in situ injection with endoscopic ultrasound, intradermal or subcutaneous vaccination, or oncolytic virus injection. In addition, majority of these trials were coupled with chemotherapy or radiochemotherapy. Only one of these trials was a phase III study, and the remaining studies were phase I or II. The phase III trial was a randomized multi-institutional study conducted to assess the efficacy of TNFerade as a novel gene transfer method to deliver tumor necrosis factor-alpha to tumor cells, in combination with 5-fluorouracil and radiotherapy for locally advanced pancreatic cancer. TNFerade was injected intratumorally before the first fraction of radiotherapy using percutaneous transabdominal or endoscopic ultrasound [37]. Although the anti-tumor effect of TNFerade was not significant, the approach was determined to be safe and confirmed to be usable in combination with the clinically well-established methods.

A summary of the currently ongoing trials can be found in Table 2 [51]. These studies include gene therapy approaches using interleukin 13 and suicide genes; gene vaccination using plasmid DNA; and oncolytic viral gene therapy for pancreatic cancer, metastatic pancreatic cancer, pancreatic neuroendocrine tumor, and pancreatitis. In addition, cell therapies, including CAR-T for adoptive immunotherapy, are being evaluated in clinical trials. This system is considered as an adoptive active immunotherapy and autologous CD8 positive T cells are extracted from the patients and modified ex vivo to recognize and kill the cancer cells expressing the tumor antigen. There are more than 20 clinical trials still recruiting the candidate for the trials and newer trials will start continuously to develop the safe and effective gene therapy for the pancreatic diseases.

In these clinical trials on pancreatic diseases, standard methods of systemic administration and in situ gene transfer by infusion or electroporation, and site-specific administration using endoscopic ultrasound techniques are combined with various chemotherapies, including gemcitabine, cyclophosphamide, fludarabine, capecitabine, cisplatin, nab-paclitaxel, docetaxel, fluorouracil, sunitinib, and everolimus. For example, the clinical trial of NCT01274455 showed the safety of administration of non-viral gene product of CYL-02, which encodes mouse somatostatin receptor subtype 2 and a fusion gene of deoxycytidine kinase and monophosphate kinase, which could sensitize the cancer cells to gemcitabine [44] and the clinical trial of NCT00836407 evaluated the effect of granulocyte-macrophage colony stimulating factor gene-delivered pancreatic tumor cells with the ipilimumab [47]. This macrophage colony stimulating factor cold also been used for the cancer vaccine therapy [46]. Majority of these trials are phase I or II, and further enrollment and assessment of the safety and efficacy is essential. However, pancreas-specific gene targeting can be achieved with the development of delivery methods, vectors, and clinical techniques. In addition, genome information-based precision medicine with mutation-targeted therapy is being tested in a phase II trial on advanced low- or intermediate-grade neuroendocrine tumors of the pancreas (NCT02315625).

## 8. Conclusions and Future Directions

Pancreas is a key organ for digestion and endocrine function. Among the diseases affecting the pancreas, pancreatic cancer is a leading cause of cancer-related deaths worldwide. With the development of studies on gene therapy, various clinical trials are ongoing and showing the promising results, as well as in the basic preclinical researches for the gene delivery. With recent advances in promising technologies, such as the generation of induced pluripotent stem cells and gene editing by CRISPR/Cas9, development of delivery systems, carriers, and methods is essential. The findings of these studies and new methodologies that are developed for gene therapy can also be useful for currently untreatable pancreatic diseases and further studies to combine these methodologies will encourage new endeavor for the novel therapeutic options. For example, as in vivo gene editing based on CRISPR/Cas9, short-term transgene expression may be preferred, in order to prevent off-target effects, therefore, transient gene expression mediated by non-viral vector-based delivery methods might be advantageous over gene editing. While further investigation is needed to improve efficacy, with the advanced gene delivery methods armed with personal genomic information, these methods can be tailored to each patient based on specific biomarkers, which should ultimately improve prognosis and extend the life expectancy of the patients with pancreatic diseases.

## Figures and Tables

**Table 1:** Summary of clinical trials published.

No.	Ref.	Conditions	Title	Carrier	Interventions	Phases	Enrollment
1	[19]	Pancreatic Cancer	Gene therapy with Adv-IL-2 in unresectable digestive cancer: phase I-II study, intermediate report.	Adenovirus	AdV/Interleukin 2	Phase 1/Phase 2	7
2	[20]	Pancreatic Cancer	Safety and feasibility of injection with an E1B-55 kDa gene-deleted, replication-selective adenovirus (ONYX-015) into primary carcinomas of the pancreas: a phase I trial.	Adenovirus	AdV/ONYX-015	Phase 1	23
3	[21]	Pancreatic Cancer	Treatment of inoperable pancreatic carcinoma using a cell-based local chemotherapy: results of a phase I/II clinical trial.	Lipofectamine (Plasmid DNA)	Lipofectamine/Cyto. P450	Phase 1/Phase 2	14
4	[22]	Pancreatic Cancer	Mucin gene (MUC1) transfected dendritic cells as vaccine: results of a phase I/II clinical trial.	Cationic liposome (Plasmid DNA)	Cationic liposome/dendritic cells transfected with MUC1 cDNA	Phase 1/Phase 2	10
5	[23]	Pancreatic Cancer	A phase I/II trial of intratumoral endoscopic ultrasound injection of ONYX-015 with intravenous gemcitabine in unresectable pancreatic carcinoma.	Adenovirus	AdV/ONYX-015 + gemcitabine	Phase 1/Phase 2	21
6	[24]	Pancreatic Cancer	First clinical experience using a ‘pathotropic’ injectable retroviral vector (Rexin-G) as intervention for stage IV pancreatic cancer.	Retrovirus	Rv/Rexin-G	Phase 1	3
7	[25]	Pancreatic Cancer	Phase I trial of intratumoral injection of an adenovirus encoding interleukin-12 for advanced digestive tumors.	Adenovirus	AdV/Interleukin 12	Phase 1	7
8	[26]	Pancreatic Cancer	TNFerade biologic, an adenovector with a radiation-inducible promoter, carrying the human tumor necrosis factor alpha gene: a phase I study in patients with solid tumors.	Adenovirus	TNFerade	Phase 1	30
9	[27]	Pancreatic Cancer	Intratumoral injection of dendritic cells engineered to secrete interleukin-12 by recombinant adenovirus in patients with metastatic gastrointestinal carcinomas.	Adenovirus	Adv encoding interleukin-12 gene	Phase 1	11
10	[28]	Pancreatic Cancer	Poxvirus-based vaccine therapy for patients with advanced pancreatic cancer.	Vaccinia and Pox virus	Vaccinia and Pox virus expressing CEA MUC-1 and co-stimulatory molecules	Phase 1	10
11	[29]	Pancreatic Cancer	Allogeneic granulocyte macrophage colony-stimulating factor-secreting tumor immunotherapy alone or in sequence with cyclophosphamide for metastatic pancreatic cancer: a pilot study of safety, feasibility, and immune activation.	Plasmid DNA	Plasmid/GVAX	Phase 1	50
12	[30]	Pancreatic Cancer	Advanced phase I/II studies of targeted gene delivery in vivo: intravenous Rexin-G for gemcitabine-resistant metastatic pancreatic cancer.	Retrovirus	Rv/Rexin-G at two dosages	Phase 1/Phase 2	9
13	[31]	Pancreatic Cancer	A phase I dose-escalation clinical trial of intraoperative direct intratumoral injection of HF10 oncolytic virus in non-resectable patients with advanced pancreatic cancer.	Herpes virus	HF10 oncolytic herpes virus	Phase 1	6
14	[32]	Pancreatic Cancer	Mutated Ras-transfected, EBV-transformed lymphoblastoid cell lines as a model tumor vaccine for boosting T-cell responses against pancreatic cancer: a pilot trial.	Immunotherapy	Lymphocytes modified with an episomal EBV expressing Ras mutant	Phase 1	7
15	[33]	Pancreatic Cancer	Phase 1/2a, dose-escalation, safety, pharmacokinetic and preliminary efficacy study of intratumoral administration of BC-819 in patients with unresectable pancreatic cancer.	Plasmid DNA	Plasmid/expression of diphtheria-toxin gene	Phase 1	9
16	[34]	Pancreatic Cancer	EUS or percutaneously guided intratumoral TNFerade biologic with 5-fluorouracil and radiotherapy for first-line treatment of locally advanced pancreatic cancer: a phase I/II study.	Adenovirus	AdV/TNFerade with chemoradiation	Phase 1	50
17	[35]	Pancreatic Cancer	A live-attenuated Listeria vaccine (ANZ-100) and a live-attenuated Listeria vaccine expressing mesothelin (CRS-207) for advanced cancers: phase I studies of safety and immune induction.	Cancer vaccine	Attenuated listeria vaccine	Phase 1	26
18	[36]	Pancreatic Cancer	Addition of algenpantucel-L immunotherapy to standard adjuvant therapy for pancreatic cancer: a phase 2 study.	Immunotherapy	Algenpantucel-L + gemcitabine + 5FU	Phase 2	70
19	[37]	Pancreatic Cancer	Randomized phase III multi-institutional study of TNFerade biologic with fluorouracil and radiotherapy for locally advanced pancreatic cancer: final results.	Adenovirus	AdV/TNFerade + Chemoradiation Vs chemoradiation	Phase 3	304
20	[38]	Pancreatic Cancer	Encapsulated cells expressing a chemotherapeutic activating enzyme allow the targeting of subtoxic chemotherapy and are safe and efficacious: data from two clinical trials in pancreatic cancer.	Lipofectamine (Plasmid DNA)	Lipofectamine/Cyto. P450	Phase 2	13
21	[39]	Pancreatic Cancer	Gene-mediated cytotoxic immunotherapy as adjuvant to surgery or chemoradiation for pancreatic adenocarcinoma.	Adenovirus	AdV/HSV thymidine kinase	Phase 1	24
22	[40]	Pancreatic Cancer	RNAi therapy targeting KRAS in combination with chemotherapy for locally advanced pancreatic cancer patients.	RNAi	SiG12-LODER^®^ + gemcitabine	Phase 1/Phase 2	15
23	[41]	Pancreatic Cancer	Safety and survival with GVAX pancreas prime and Listeria Monocytogenes-expressing mesothelin (CRS-207) boost vaccines for metastatic pancreatic cancer.	Cancer vaccine	GVAX + CRS 2017	Phase 2	90
24	[42]	Pancreatic Cancer	Randomized Phase 2 Trial of the Oncolytic Virus Pelareorep (Reolysin) in Upfront Treatment of Metastatic Pancreatic Adenocarcinoma.	Reovirus	Reolysin + paclitaxel + carboplatin	Phase 2	73
25	[43]	Pancreatic Cancer	AdV/Theragene + Chemotherapy	Adenovirus	AdV/Theragene + Chemotherapy	Phase 1	9

**Table 2:** Summary of clinical trials ongoing.

No.	Ref.	NCT Number	Conditions	Title	Carrier	Phases	Enrollment	Study Start	Study Completion
1		NCT00415454	Pancreatic Cancer	Study Combining Suicide Gene Therapy with Chemoradiotherapy in the Treatment of Non-Metastatic Pancreatic Adenocarcinoma	Adenovirus	Phase 1	8	November 2006	N/A
2	[44]	NCT01274455	Pancreatic Cancer	Gene Therapy of Pancreatic Ductal Adenocarcinoma	Plasmid DNA	Phase 1	22	December 2010	March 2013
3		NCT02806687	Pancreatic Cancer	Effect of Intratumoral Injection of Gene Therapy for Locally Advanced Pancreatic Cancer	Plasmid DNA	Phase 2	100	January 2017	June 2019
4		NCT02894944	Pancreatic Cancer	Clinical Trial Phase I for Theragene in Combination with Chemotherapy for the Locally Advanced Pancreatic Cancer	Adenovirus	Phase 1	9	August 2016	July 2018
5	[45]	NCT00121745	Pancreatic Cancer	Evaluation of Safety of Rexin-G Gene Transfer for Advanced Pancreatic Cancer	Retrovirus	Phase 1	12	July 2005	July 2007
6		NCT03165188	Pancreatic Cancer	Long Term Follow-Up Study for Subjects Previously Treated With Algenpantucel-L (HyperAcute-Pancreas) Immunotherapy	Immunotherapy	Not Applicable	500	September 2017	June 2031
7		NCT01583686	Pancreatic Cancer	CAR T Cell Receptor Immunotherapy Targeting Mesothelin for Patients With Metastatic Cancer	CAR-T	Phase 1/Phase 2	136	April 2012	December 2028
8		NCT02830724	Pancreatic Cancer	Administering Peripheral Blood Lymphocytes Transduced With a CD70-Binding Chimeric Antigen Receptor to People With CD71 Expressing Cancers	CAR-T	Phase 1/Phase 2	113	April 2017	January 2028
9		NCT00638612	Pancreatic Cancer	AdV-tk Therapy With Surgery and Chemoradiation for Pancreas Cancer (PaTK02)	Adenovirus	Phase 1	27	August 2008	June 2015
10		NCT02465983	Pancreatic Cancer	Pilot Study of Autologous T-cells in Patients With Metastatic Pancreatic Cancer	CAR-T	Phase 1	4	May 2015	November 2017
11		NCT03190941	Pancreatic Cancer	Administering Peripheral Blood Lymphocytes Transduced With a Murine T-Cell Receptor Recognizing the G12V Variant of Mutated RAS in HLA-A*1102 Patients	Immunotherapy	Phase 1/Phase 2	110	September 2017	June 2028
12		NCT03225989	Pancreatic Cancer	Trial Investigating an Immunostimulatory Oncolytic Adenovirus for Cancer	Adenovirus	Phase 1/Phase 2	50	March 2018	December 2022
13		NCT03192462	Pancreatic Cancer	TAA Specific Cytotoxic T Lymphocytes in Patients With Pancreatic Cancer	Immunotherapy	Phase 1/Phase 2	45	January 2018	November 2025
14		NCT00004178	Pancreatic Cancer	Gene Therapy in Treating Patients With Cancer	Immunotherapy	Phase 1	null	April 1998	December 2001
15	[46]	NCT00084383	Pancreatic Cancer	Vaccine Therapy Combined With Adjuvant Chemoradiotherapy in Treating Patients With Resected Stage I or Stage II Adenocarcinoma (Cancer) of the Pancreas	Cancer vaccine	Phase 2	60	January 2002	July 2006
16	[47]	NCT00836407	Pancreatic Cancer	Ipilimumab +/- Vaccine Therapy in Treating Patients With Locally Advanced, Unresectable or Metastatic Pancreatic Cancer	Cancer vaccine	Phase 1	30	February 2009	July 2012
17		NCT00305760	Pancreatic Cancer	Vaccine Therapy, Cyclophosphamide, and Cetuximab in Treating Patients With Metastatic or Locally Advanced Pancreatic Cancer	Cancer vaccine	Phase 2	60	December 2005	N/A
18		NCT02750657	Pancreatic Cancer	Study of Changes and Characteristics of Genes in Patients With Pancreatic Cancer for Better Treatment Selection	Genetic Profiling	Not Applicable	180	December 2015	December 2021
19		NCT00303927	Pancreatic Cancer	Capecitabine as Second-Line Therapy in Treating Patients With Stage IV Pancreatic Cancer Who Have the Thymidylate Synthase Gene	Genetic Profiling	Phase 2	65	December 2005	N/A
20		NCT01188109	Pancreatic Cancer	Gemcitabine/Cisplatin for Resected Pancreas Cancer: Establishing the Role of ERCC2 in Treatment Decision	Genetic Profiling	Phase 2	25	July 2010	July 2015
21		NCT00389610	Pancreatic Cancer	Vaccine Therapy in Treating Patients With Pancreatic Cancer That Has Been Removed by Surgery	Cancer vaccine	Phase 2	56	September 2006	December 2018
22		NCT01394120	Pancreatic Cancer	Chemotherapy Selection Based on Therapeutic Targets for Advanced Pancreatic Cancer	For Targeted and Tailored Treatment	Phase 2	60	August 2011	December 2013
23		NCT00066404	Pancreatic Cancer	Intrapleural BG00002 in Treating Patients With Malignant Pleural Mesothelioma or Malignant Pleural Effusions	Recombinant adenovirus	Phase 1	null	April 2003	N/A
24		NCT01474564	Pancreatic Cancer	Feasibility of Obtaining and Characterizing Circulating Tumorigenic Cells in Patients With Pancreatic Adenocarcinoma	Genetic Profiling	Not Applicable	60	November 2011	November 2019
25		NCT02405585	Pancreatic Cancer	Immunotherapy and SBRT Study in Borderline Resectable Pancreatic Cancer	Immunotherapy	Phase 2	10	April 2015	N/A
26		NCT02705196	Pancreatic Cancer	LOAd704 Oncolytic Virus Therapy for Pancreatic Cancer	Adenovirus	Phase 1/Phase 2	26	November 2016	August 2019
27		NCT00669734	Pancreatic Cancer	Vaccine Therapy and Sargramostim in Treating Patients With Pancreas Cancer That Cannot Be Removed By Surgery	Cancer vaccine	Phase 1	18	February 2010	N/A
28		NCT00727441	Pancreatic Cancer	Vaccine Therapy With or Without Cyclophosphamide in Treating Patients Undergoing Chemotherapy and Radiation Therapy for Stage I or Stage II Pancreatic Cancer That Can Be Removed by Surgery	Cancer vaccine	Not Applicable	87	July 2008	March 2018
29		NCT00947102	Pancreatic Cancer	Influence of Gemcitabine Treatment on Immunological and Serological Profile in Patients With Pancreatic Cancer	Observation	Not Applicable	null	February 2009	December 2011
30		NCT00051467	Pancreatic Cancer	A Study of TNFerade Biologic With 5-FU and Radiation Therapy for First-Line Treatment of Unresectable Locally Advanced Pancreatic Cancer	Adenovirus	Phase 3	null	N/A	N/A
31		NCT03531125	Pancreatic Cancer	Gene Expression in Resectable Pancreatic Cancer	Procedure of Endoscopic Ultrasound	Not Applicable	10	June 2018	December 2019
32		NCT00429858	Pancreatic Cancer	Gemcitabine and S-2 for Locally Advanced Unresectable or Metastatic Pancreatic Cancer	Chemotherapy	Phase 2	21	January 2007	October 2010
33		NCT02568267	Pancreatic Cancer	Basket Study of Entrectinib (RXDX-101) for the Treatment of Patients With Solid Tumors Harboring NTRK 1/2/3 (Trk A/B/C), ROS2, or ALK Gene Rearrangements (Fusions)	Genetic Profiling	Phase 2	300	November 2015	October 2020
34		NCT00159471	Pancreatic Cancer	Genes as Predictors of Response to Gemcitabine, Docetaxel, and Capecitabine (GTX) in Metastatic or Unresectable Pancreatic Cancer.	Genetic Profiling	Not Applicable	1	February 2005	July 2006
35		NCT00386399	Pancreatic Cancer	Study of Mitomycin-C in Patients With Advanced or Recurrent Pancreatic Cancer With Mutated BRCA3 Gene	Genetic Profiling	Phase 2	0	October 2006	February 2008
36		NCT01836432	Pancreatic Cancer	Immunotherapy Study in Borderline Resectable or Locally Advanced Unresectable Pancreatic Cancer	Immunotherapy	Phase 3	302	May 2013	June 2017
37		NCT00255827	Pancreatic Cancer	Vaccine Treatment for Surgically Resected Pancreatic Cancer	Cancer vaccine	Phase 1/Phase 2	7	November 2005	September 2007
38		NCT01938716	Pancreatic Cancer	Gemcitabine Pharmacokinetics After Preoperative Chemoradiation Therapy	Chemotherapy	Not Applicable	40	March 2012	March 2019
39		NCT03193190	Pancreatic Cancer	A Study of Multiple Immunotherapy-Based Treatment Combinations in Participants With Metastatic Pancreatic Ductal Adenocarcinoma (Morpheus-Pancreatic Cancer)	Immunotherapy	Phase 1/Phase 2	185	July 2017	September 2020
40		NCT00089024	Pancreatic Cancer	Combination Chemotherapy, and Radiation Therapy in Treating Patients With Locally Advanced Pancreatic Cancer	Chemotherapy	Phase 2	50	February 2004	N/A
41		NCT02465060	Pancreatic Cancer	Targeted Therapy Directed by Genetic Testing in Treating Patients With Advanced Refractory Solid Tumors, Lymphomas, or Multiple Myeloma (The MATCH Screening Trial)	Genetic Profiling	Phase 2	6452	August 2015	N/A
42		NCT01191684	Pancreatic Cancer	Vaccine Therapy in Treating Patients With Colorectal, Stomach, or Pancreatic Cancer	Cancer vaccine	Phase 1	12	October 2011	August 2013
43		NCT01088789	Pancreatic Cancer	A Trial of Boost Vaccinations of Pancreatic Tumor Cell Vaccine	Plasmid DNA	Phase 2	72	April 2010	April 2023
44		NCT02514421	Pancreatic Cancer	Evaluation of Safety and Efficacy of Electrochemotherapy in the Treatment of Pancreatic Adenocarcinoma	Device of Electroporation	Not Applicable	24	July 2015	July 2017
45		NCT02414100	Pancreatic Cancer	Patient Derived Cancer Cell Lines in Identifying Molecular Changes in Patients With Previously Untreated Pancreatic Cancer Receiving Gemcitabine Hydrochloride-Based Chemotherapy	Genetic Profiling	Not Applicable	0	December 2013	December 2016
46		NCT00936104	Pancreatic Cancer	Side Population in Pancreatic Ductal Adenocarcinoma (PDAC)		Not Applicable	20	August 2008	July 2012
47		NCT03302637	Pancreatic Cancer	Oral Microbiome and Pancreatic Cancer	Genetic Profiling	Not Applicable	732	December 1992	December 2010
48		NCT03602079	Pancreatic Cancer	Study of A166 in Patients With Relapsed/Refractory Cancers Expressing HER2 Antigen or Having Amplified HER3 Gene	Genetic Profiling	Phase 1/Phase 2	82	July 2018	May 2021
49		NCT03337087	Pancreatic Cancer	Liposomal Irinotecan, Fluorouracil, Leucovorin Calcium, and Rucaparib in Treating Patients With Metastatic Pancreatic, Colorectal, Gastroesophageal, or Biliary Cancer	Chemotherapy	Phase 1/Phase 2	110	August 2018	December 2022
50		NCT00128622	Pancreatic Cancer	Denileukin Diftitox Followed by Vaccine Therapy in Treating Patients With Metastatic Cancer	Cancer vaccine	Phase 1	24	September 2005	May 2009
51		NCT02592395	Pancreatic Cancer	Study of FOLFIRINOX Electrochemotherapy in the Treatment of Pancreatic Adenocarcinoma	Device of Electroporation	Phase 1	0	October 2015	October 2017
52		NCT02432963	Pancreatic Cancer	Vaccine Therapy and Pembrolizumab in Treating Patients With Solid Tumors That Have Failed Prior Therapy	Cancer vaccine	Phase 1	19	November 2015	February 2019
53		NCT00959946	Pancreatic Cancer	Study Of Bosutinib With Capecitabine In Solid Tumors And Locally Advanced Or Metastatic Breast Cancer	Chemotherapy	Phase 1/Phase 2	32	September 2009	March 2011
54		NCT01643499	Pancreatic Cancer	Genotype-guided Dosing of mFOLFIRINOX Chemotherapy in Patients With Previously Untreated Advanced Gastrointestinal Malignancies	Genetic Profiling	Phase 1	79	March 2012	August 2020
55		NCT02576665	Pancreatic Cancer	A Study of Toca 511, a Retroviral Replicating Vector, Combined With Toca FC in Patients With Solid Tumors or Lymphoma (Toca 7)	Retrovirus	Phase 1	30	July 2016	November 2019
56		NCT00711997	Pancreatic Cancer	Phase 1/2a DTA-H19 in Patients With Unresectable Pancreatic Cancer	Plasmid DNA	Phase 1/Phase 2	9	August 2009	December 2010
57		NCT02239861	Pancreatic Cancer	TAA-Specific CTLS for Solid Tumors (TACTASOM)	Immunotherapy	Phase 1	16	April 2015	December 2018
58		NCT03281382	Metastatic Pancreatic Cancer	Phase 1 Trial of Interleukin 13 Gene Therapy for Metastatic Pancreatic Cancer	Adenovirus	Phase 1	9	July 2017	June 2021
59		NCT02340117	Metastatic Pancreatic Cancer	Study of Combined SGT-54 Plus Gemcitabine/Nab-Paclitaxel for Metastatic Pancreatic Cancer	Immunotherapy	Phase 2	28	January 2015	December 2020
60		NCT00868114	Metastatic Pancreatic Cancer	Direct Tumor Injection KLH-Pulsed Dendritic Cells in Unresectable Pancreatic Cancer	Cell	Phase 2	35	July 2006	December 2015
61		NCT01437007	Metastatic Pancreatic Cancer	TKM 080302 for Primary or Secondary Liver Cancer	RNAi	Phase 1	1	August 2011	June 2012
62		NCT02416466	Metastatic Pancreatic Cancer	CAR-T Hepatic Artery Infusions and Sir-Spheres for Liver Metastases	CAR-T	Phase 1	8	April 2015	January 2019
63		NCT01116791	Peritoneal Carcinomatosis	Cytoreductive Surgery (CRS) Plus Hyperthermic Intraoperative Peritoneal Chemotherapy(HIPC) With Cisplatin to Treat Peritoneal Carcinomatosis From Upper Gastrointestinal Cancer	Procedure of Cytoreductive Surgery plus Hyperthermic Intraoperative Peritoneal Chemotherapy	Phase 2	34	July 2010	December 2015
64		NCT02315625	Neuroendocrine Tumors of the Pancreas	Study of Mutation-Targeted Therapy With Sunitinib or Everolimus in People With Advanced Low- or Intermediate-Grade Neuroendocrine Tumors of the Gastrointestinal Tract and Pancreas With or Without Cytoreductive Surgery	Genetic Profiling	Phase 2	120	April 2015	December 2025
65		NCT00444444	Pancreatitis	Genetic Analysis for Predicting of Relapse During Steroid Treatment for Autoimmune Pancreatitis (AIP)			40	February 2002	June 2007

**Table 3:** Gene delivery methods used in clinical and pre-clinical stages.

Gene Transfer Methods	Functional Component	Targeted Genes	Features	Features
**Viral Vectors**		Tumor suppressor genes, Pro-apoptotic genes, Suicide genes, siRNA, miRNA,		
Oncoretrovirus	RNA		High efficiency	Random integration, low titer
Lentivirus	RNA		High efficiency, sustained gene expression	Random integration, low titer
Foamy virus	RNA		High efficiency, sustained gene expression	Random integration, low titer
Adenovirus	Double stranded DNA		High efficiency, sustained gene expression, infect non-dividing cells	Host innate immune response
Adeno-associated virus	Single stranded DNA		No pathogenic, sustained gene expression, infect to non-dividing cells	Integration may occur, small capacity of transgene, low titer
Herpes simplex virus	Double stranded DNA		No integration, sustained gene expression	Low transduction efficiency
**Non-viral Vectors (Chemicals)**				
Lipids	Cationic lipids		High efficiency in vitro, ease to prepare	Low efficiency in vivo, acute immune response
Polymers	Cationic polymers		Highly effective in vitro, ease to prepare	Toxic to cells, acute immune response
Proteins	Natural or chemically modified proteins in cationic nature		Highly effective in vitro, less toxic, can be target specific	Low activity in vivo
Peptides	Lysine or arginine residues in peptides		Highly effective in vitro, less toxic, can be target specific	Low activity in vivo
**Non-viral Vectors (Physical Methods)**				
Needle injection	Mechanic force		Simple	Low efficiency, expression limited to needle track
Gene gun	Pressure		Good efficiency	Limited to target area, need surgical procedure for internal organ
Electroporation	Electric pulse		High efficiency	Tissue damage, limited target area, need surgical procedure for internal organ
Sonoporation	Ultrasound		Site specific	Low efficiency, tissue damage
Magnetofection	Magnetic field		Site specific	Low efficiency, limited target area, need surgical procedure for internal organ
Hydrodynamic delivery	Hydrodynamic pressure		Simple, high efficiency, site specific	Need catheter insertion technique in large animals
**Immunotherapy**	Cytokines		Require ex vivo cell culture	
**Adoptive Immunotherapy**	CAR-T		Require ex vivo cell culture	
**Vaccination**	Antigen-pulsed dendritic cells		Intravenous or subcutaneous or local administration	
